# Increased common corticospinal input during eyes-closed unilateral stance in people with chronic ankle instability

**DOI:** 10.1038/s41598-026-39425-3

**Published:** 2026-02-12

**Authors:** Xiaohan Xu, Joanna Bowtell, William R. Young, Daniel T. P. Fong, Genevieve K. R. Williams

**Affiliations:** 1 Public Health and Sports Sciences Department, University of Exeter, Exeter, EX1 2LU UK; 2 National Centre for Sport and Exercise Medicine, School of Sport, Exercise and Health Sciences, Loughborough University, Loughborough, LE11 3TT UK

**Keywords:** Chronic ankle instability, Intermuscular coherence, Corticospinal input, Muscle coordination, Neuromuscular control, Rehabilitation, Motor control, Sensorimotor processing, Sensory processing

## Abstract

Neuromuscular control deficits and altered spinal and corticospinal mechanisms are central to chronic ankle instability (CAI) and its persistent symptoms, but the role of ankle muscle coordination and common neural inputs during unipedal stance in CAI remains unexplored. This study aimed to compare intermuscular coherence between individuals with CAI and healthy controls during single-leg stance and investigate functionality of intermuscular coherence to postural control. Sixteen CAI and 16 healthy control (HC) participants performed single-leg balance tasks under eyes-open and eyes-closed conditions. The surface electromyograms were recorded from tibialis anterior (TA), peroneus longus (PL), gastrocnemius medial head (GM), and soleus (SOL) muscles. Coherence was analysed for PL-TA, PL-SOL, PL-GM, SOL-TA and SOL-GM muscle pairs in the delta (0.5–5 Hz) and beta (15–35 Hz) frequency bands. The CAI group exhibited greater beta-band intermuscular coherence for PL-SOL, PL-GM and SOL-TA during eyes-closed stance but not in eyes-open conditions, compared to healthy controls, suggesting increased common corticospinal inputs to agonist-antagonist muscle pairs. Higher beta-band coherence in the antagonistic muscle pairs correlated with reduced COP complexity, suggesting that strengthened beta-band indicates reduced postural adaptability. These findings suggest increased common corticospinal inputs to agonist-antagonist muscle pairs in CAI individuals, suggesting reduced postural adaptability during eyes-closed stance. Future research should address methodological considerations and validate protocol for intermuscular coherence analysis during single-leg stance. Future studies should also include CAI copers to determine whether their common corticospinal inputs have returned to healthy levels, supporting the potential effectiveness of targeted neuromodulatory or rehabilitation interventions.

**Supplementary Information:**

The online version contains supplementary material available at 10.1038/s41598-026-39425-3.

## Introduction

Lateral ankle sprains are one of the most prevalent musculoskeletal injuries in both athletic and general populations^1^. Epidemiological studies have indicated that approximately 70% of the general population experience an ankle sprain during their lifetime^2^. These injuries cause acute functional impairment and have long-term effects, with nearly 50% of ankle sprains progressing to chronic ankle instability (CAI)^3^. CAI is characterised by persistent mechanical and functional instability, affecting neuromuscular control and increasing the risk of recurrent injury^4^.

Diminished somatosensation in individuals with CAI results from damage to ligaments, articular receptors, and muscle spindles, leading to secondary deafferentation through inflammation and effusion^5^. Proprioception deficits have been reported in joint position sense^6^, force sense^7^ and cutaneous sensation^8^. Additionally, individuals with CAI demonstrate a heavier reliance on visual inputs over somatosensory during single-leg stance, suggesting impaired multisensory integration^9^. The reliance on visual feedback implies an inability to emphasise somatosensory inputs to the same extent as healthy people^10^, yet the neurophysiological basis for the impairment remains unclear.

Neuromuscular control impairment has been found in people with CAI, particularly in peroneus longus (PL) function, compared to healthy controls. Studies have reported inconsistent findings in EMG amplitude and timing during walk^11,12^, no difference during running^13^, reduced activation during cutting^14^, or no difference in cutting tasks^15,16^, and reduced PL activation during pre-impact phase of landing^17^. Combined with delayed and reduced PL responses to sudden inversion perturbations^18^, these findings indicate impairments in sensorimotor control, likely due to deficits in afferent input, central or spinal processing and/or motor response. Reduced spinal reflex excitability, evidenced by lower Hmax/Mmax ratios in the PL and soleus muscles^19^, points to significant spinal contribution in CAI. Supraspinal mechanisms operating in CAI remain controversial, some reported increased motor thresholds of PL, suggesting reduced excitability^20^, while others did not^21^; More consistent reports are observed for decreased motor-evoked potential amplitudes^22^, possibly reflecting reduced corticomotor area or volume mapped to the PL^23^. Increased intracortical inhibition has been demonstrated in CAI individuals, suggested by prolonged cortical silent periods^24^, though not all studies confirm this, and there is debate about whether the cortical silent period reliably indicates cortical inhibition^25^. Diffusion tensor imaging further indicates reduced connectivity in the superior cerebellar peduncle^26^. Efforts to modulate spinal excitability through interventions such as cooling, manual therapy, taping, and vibration have shown mixed effectiveness^27^. These sensorimotor impairments in CAI may stem from reduced spinal reflex excitability, inhibited central processing and motor response, highlighting the need to understand spinal and supraspinal mechanisms for effective targeted interventions.

Adequate ankle stiffness is essential for maintaining upright postural control, provided by the muscle-tendon complex and surrounding tissue^28^. In individuals with CAI, increased talocrural joint laxity can impair mechanical stability, which might lead to a compensatory strategy of muscle co-contraction to regulate ankle stiffness. However, findings on PL-TA co-contraction levels in CAI are mixed; CAI individuals exhibited either no difference^29,30^, increased^31^ or decreased co-contraction^32^, compared to healthy controls during single-leg landing. Variability in electromyography (EMG) normalisation due to inconsistent maximal voluntary contractions, affected by task instructions (e.g. repetitions, hold stable) or inability to achieve maximal contraction, may explain the discrepancies in findings^33^. The frequency analysis of surface EMG is a useful method for the study of the neuromuscular system, mostly used to test the myoelectric features of muscle fatigue^34^, and quantify coherence between signals^35^.

Intermuscular coherence (IMC) is time and frequency domain analysis of the coupling between pairs of EMG signals of two muscles^35^. The EMG signal contains spectral information on motor neuron firing and trains of action potentials, where common synaptic inputs to motor neuron pools in multiple muscles can synchronise their firing frequencies^36^. Different frequency inputs to muscles indicate activity from specific brain regions and circuits, enabling identification of presynaptic common inputs (spinal, subcortical or corticospinal) based on coherence frequencies^37^. In general, delta-band coherence (0–5 Hz) reflects neural drive to muscle and is related to force production^38^, and beta-band coherence (15–35 Hz) is related to corticospinal drive^37^. Although the ongoing research challenges that surface EMG does not directly reflect motor unit recruitment^34^, it provides global information on muscle activity and may be useful for examining intermuscular synchronisation at the whole-muscle level. Coherence values are nondeterministic, as their magnitude might depend on task demands and pathological conditions, reflecting different neuromuscular control strategies. For example, increased beta-band coherence has been observed in older adults during standing and walking^39,40^ and in Parkinson’s disease during isometric leg contraction^41^, suggesting an adaptive corticospinal response; Delta-band increases with greater balance challenges^42,43^. However, no research to date has investigated the IMC in people with CAI, and its potential functionality to postural control remains unclear. The IMC provides insights into spinal and corticospinal control, so the investigation of clinically important muscles, such as peroneus longus and soleus, to their agonist and antagonist muscles (e.g. tibialis anterior and gastrocnemius) is warranted.

To better understand neuromuscular strategies used by people with CAI, the study aims to identify IMC differences in individuals with CAI compared to healthy controls (HC) during single-leg stance and assess how visual input affects these coherence patterns. The second aim is to investigate association between IMC and postural sway (i.e., centre of pressure fractal dimension, COP-FD), seeking to indicate potential functionality of IMC. We hypothesized that (1) individuals with CAI would show lower delta-band coherence and higher beta-band coherence during eyes-closed stance; (2) IMC would associate with postural control measures.

## Methods

### Participants

Sixteen participants with CAI and 16 healthy controls participated in the study after providing written informed consent. This is the first study investigating EMG-EMG IMC in individuals with CAI. An a priori power analysis was conducted based on previous studies comparing young and older adults during single-leg stance with eyes open and eyes closed^44^. As both older adults and individuals with CAI experience postural instability during single-leg balance tasks, we utilized the effect sizes from these studies to estimate the sample size for the current investigation. With an effect size Cohen’s f of 0.264^45^, power of 0.8, and α = 0.05, at least 16 participants per group are needed to detect a significant interaction effect in a 2 × 2 repeated-measures ANOVA. Participants were instructed about the study purpose and procedures and signed written informed consent prior to participating. The Sports and Health Sciences Department Ethics Committee approved the study (1071581). All methods were performed in accordance with the relevant guidelines and regulations.

Both groups were similar in gender, age, height, body mass, and physical activity level, assessed by the International Physical Activity Questionnaire-Short Form (IPAQ-SF) (Table 1). The CAI group met the International Ankle Consortium’s criteria^46^ and a Cumberland Ankle Instability Tool score < 24 indicated impaired ankle function^47^. Control group inclusion required no history of lateral ankle sprains. All participants were aged 18–35 years and had no acute musculoskeletal injury within three months prior to testing, nor any history of visual or hearing disorders, dizziness, recurrent falls, vestibular dysfunction, lower extremity fractures, pain, surgery, or professional balance training experience.

**Table 1:** Participants’ demographics and anthropometrics.

	CAI (*n* = 16)	HC (*n* = 16)
Sex	Female – 8	Female – 7
	Male – 8	Male – 9
Age, years - mean ± SD	22.2 ± 1.9	23.3 ± 2.9
Height, m - mean ± SD	1.73 ± 0.08	1.72 ± 0.07
Body Mass, kg - mean ± SD	75.9 ± 13.5	68.4 ± 8.7
IPAQ-SF	Moderate − 9	Moderate – 9
	High − 7	High – 7
CAIT Score	17.3 ± 3.4	30.0 ± 0

SD: standard deviation; IPAQ-SF: International Physical Activity Questionnaire-Short Form CAIT: Cumberland Ankle Instability Tool.

### Instruments and procedures

This cross-sectional observational study investigated IMC and postural control in individuals with CAI and HC under eyes-open (EO) and eyes-closed (EC) conditions. The independent variables were group (CAI vs. HC) and condition (EO vs. EC), while the primary dependent variables included mean IMC for five muscle pairs (PL-TA, PL-SOL, PL-GM, SOL-TA, SOL-GM) in the delta (0.5–5 Hz) and beta (15–35 Hz) frequency bands. The delta and beta bands were analysed only, because alpha-band coherence is affected by mixed subcortical and cortical influences, lacks consistent frequency definitions^39^, and shows overlapping yet less significant functional roles compared to the delta band^48–50^, all of which increase heterogeneity and complicate result interpretation. Secondary dependent variables included COP-FD, significant coherence share, and coherence phase category, with statistical analysis focusing on mean coherence for each muscle pair and COP-FD.

Participants were required to perform single-leg balance tests barefoot on the affected side in CAI. For participants with a history of bilateral ankle sprains, the more affected side, determined by the lower CAIT score, was tested. Height-, gender-, and IPAQ-SF-matched HCs were tested on the corresponding side, and we assumed a similar distribution of leg dominance across both groups. While leg dominance may influence balance strategies, a recent review suggests the effect is not significant^51^. Balance was tested under eyes-open (EO) and eyes-closed (EC) conditions, with each trial lasting a minimum of 20 s and repeated three times. Participants were instructed to keep the standing foot pointed straight ahead, with the lifted leg held elevated, maintaining approximately 20° hip flexion and 45° knee flexion. Hands were placed on the waist, and the lifted leg was to avoid contact with the standing leg. Participants were directed to focus their gaze on a target positioned at eye level, approximately three meters away on a wall. They were also instructed to minimise unnecessary movements (e.g., scratching) to ensure consistency. In case of failure, the task was repeated until three complete measures were obtained. A trial was considered unsuccessful if balance was lost, the standing foot stepped or jumped, frictional movements occurred on the platform, as well as visually apparent movements of the lifted legs and arms beyond minor adjustments. The middle 10 s of each successful trial were analysed. Building on previous single-leg balance task under eyes-open and eyes-closed conditions^52–55^, and EMG-based research in CAI population^56,57^, 10-s window analysis was considered sensitive for detecting balance deficits in CAI.

### Data collection

Tibialis Anterior (TA), Peroneus Longus (PL), Gastrocnemius medial head (GM), and Soleus (SOL) activation were recorded using wireless EMG electrodes (size – 37 × 27 × 13 mm, Trigno, DELSYS, Boston, MA, USA; 1925.925-Hz sampling rate) and Trigno Discover software. EMG electrodes were placed following SENIAM guidelines, with particular care taken to place the PL electrode away from the edges of the TA muscle to minimise potential crosstalk^58^. Skin preparation involved abrasion using Nuprep^®^ Gel (Weaver and Company, US) and cleaning with alcohol. Baseline noise root mean square (RMS < 15µV) was assessed during sensor setup to ensure an adequate signal-to-noise ratio (SNR > 10)^59^. The SNR was calculated using the formula: SNR = 20log (RMS of signal during muscle contraction / RMS of baseline noise) by asking participants to contract their muscles. EMG signals were amplified (signal bandwidth of 20–450 Hz) via a bio-amplifier (Trigno Wireless System). Along with the EMG signals, ground reaction force and moment were collected with the AMTI force plates system (AMTI, Advanced Mechanical Technology Inc., size 120 cm by 120 cm), sampled at 1 kHz. The force plate data were digitally filtered using a fourth-order, 10 Hz low-pass Butterworth filter. All the data were processed in MATLAB 2022b (MathWorks, Inc., Natick, Massachusetts).

### Postural sway

The COP-FD was computed based on the formula^60^:

$$\:\mathrm{FRACTAL\:DIMENSION}=\frac{\mathrm{log}N}{\mathrm{log}N+\mathrm{log}\sqrt{\frac{4}{{\uppi\:}}\times\:\mathrm{95\%\:CONF.\:AREA}}-\mathrm{log}\text{SWAY \:LENGTH}}$$

$\:N$ denotes the number of points of the signal; $\:\mathrm{95\%\:CONF.\:AREA}$ represents 95% confidence ellipse area; $\:\mathrm{SWAY\:LENGTH}$ is COP total path length. The COP FD was chosen to quantify postural sway as it provides a functionally relevant metric for interpreting coherence, capturing non-linear properties of sway, with both fast and slow postural fluctuations. Building on the work of Doherty, Bleakley, Hertel, Caulfield, Ryan, Sweeney, Patterson and Delahunt^61^, who showed reduced FD, in CAI and acute ankle sprain^62^ compared to HCs, suggesting decreased complexity and adaptability to changing environments, aligning with coherence findings that reflect motoneuron pool variability and transmission efficiency.

### Wavelet coherence analysis

**Fig. 1:**
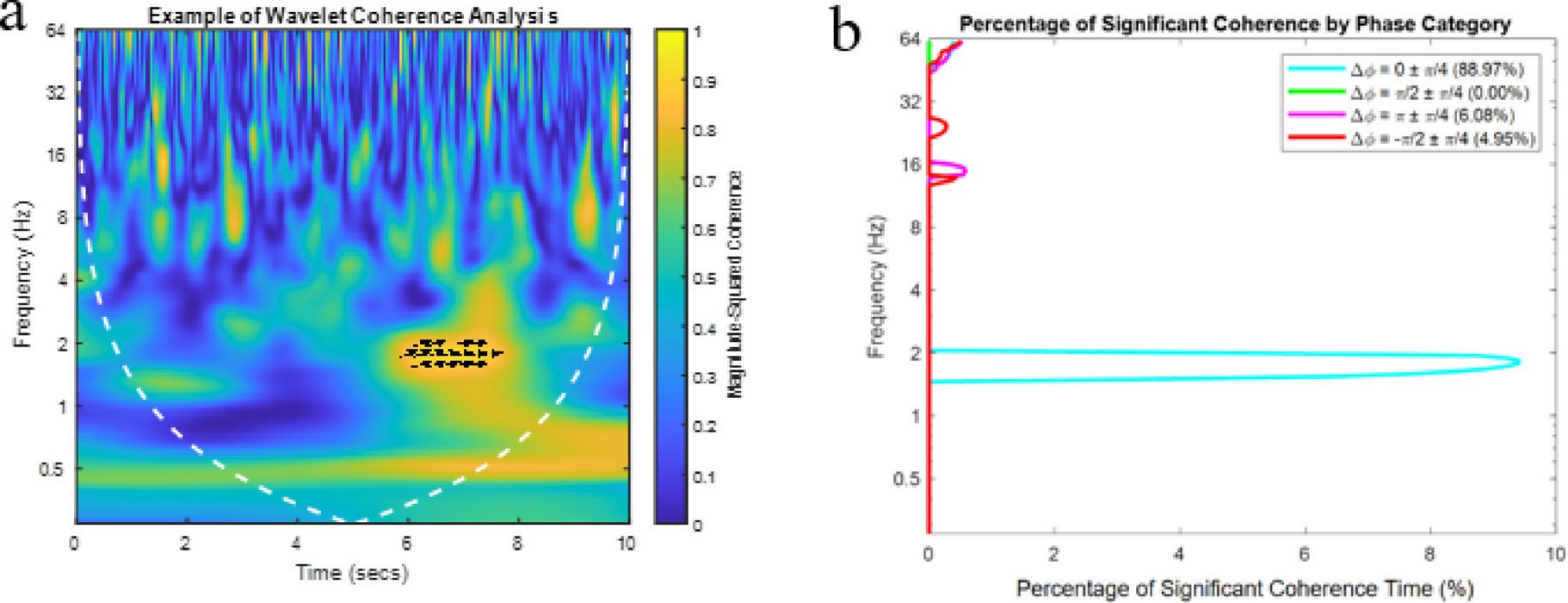
Results in wavelet coherence analysis of SOL-TA from a participant: (**a**) Wavelet coherence analysis R _SOL-TA_^2^(n, s), from a participant in the healthy control group. The x-axis represents time, the y-axis represents frequency on a logarithmic scale, and the colour scale is the magnitude of coherence. The dashed white line outlines the cone of influence (COI), where edge effects were excluded in the current analysis. Arrows indicate the significant coherence (R ^2^ > 0. 8534) and phase relationship between signals at various time-frequency points, with rightward arrows representing in-phase (Δφ = 0) coherence, and leftward arrows indicating anti-phase (Δφ = π) coherence. (**b**) The percentage of significant coherence, P(s), was quantified within four distinct phase ranges: Δφ = 0 ± π/4 (cyan), π/2 ± π/4 (green), π ± π/4 (purple), and − π/2 ± π/4 (red). For each frequency (y-axis), P(s) represents the percentage of time during which coherence values across all frequencies exceeded the significance threshold (R ^2^ > 0. 8534). The cumulative percentage for each phase category was indicated in the legend.

The raw data were visually inspected to ensure no apparent disruption or excessive noise, and all signals met quality standards for inclusion in the analysis. The EMG signals were then full wave rectified^63^. Wavelet coherence is better suited than Fourier coherence for analysing the non-stationary signals recorded during eyes-closed single-leg stance. The cross-wavelet transform of two time series, $\:\mathrm{x}\left(\mathrm{n}\right)$ and $\:\mathrm{y}\left(\mathrm{n}\right)$, is defined as:

$$\:{W}^{XY}\left(\mathrm{n},\mathrm{s}\right)={W}^{X}\left(\mathrm{n},\mathrm{s}\right){W}^{Y}\left(\mathrm{n},\mathrm{s}\right).$$

where$\:\:{W}^{X}\left(\mathrm{n},\mathrm{s}\right)$ and $\:{W}^{Y\mathrm{*}}\left(\mathrm{n},\:\mathrm{s}\right)$ denote the continuous wavelet transforms of $\:\mathrm{x}$ and $\:\mathrm{y}$ at a time index $\:\mathrm{n}$ and time scale $\:\mathrm{s}$ that is in inverse proportion to frequency, and $\:\mathrm{*}$ indicates the complex conjugate. A magnitude-squared cross wavelet coherence *R* ^2^(n, s) is defined^64^ as:

$$\:{\mathrm{R}}^{2}\left(\mathrm{n},\:\mathrm{s}\right)\:=\:\frac{{\left|\:\mathrm{S}\:\left[{\mathrm{S}}^{-1}\:{\mathrm{W}}^{\mathrm{X}\mathrm{Y}}\left(\mathrm{n},\:\mathrm{s}\right)\:\right]\right|}^{2}}{S\left[{S}^{-1}{\left|{W}^{X}\left(n,s\right)\right|}^{2}\cdot\:S{S}^{-1}{\left|{W}^{Y}\left(n,s\right)\right|}^{2}\right]}$$

where $\:{R}^{2}\left(n,s\right)$, with values ranging from 0 to 1, can be interpreted as a localised correlation coefficient between $\:\mathrm{x}\left(n\right)$ and $\:\mathrm{y}\left(n\right)$ within the time-frequency domain, and S is a smoothing operator in the time and scale. In this study, we used a MATLAB-based software package^65^ for wavelet coherence analysis between the spontaneous oscillations of EMG signals. This software package employs a Morlet wavelet (with Ω_0_ = 6) as the mother wavelet, with 24 voices per octaves, and smoothing scale of 24 used in analysis. The statistical significance of $\:{R}^{2}\left(n,s\right)$ could be estimated through Monte Carlo simulations based on a stochastic Gaussian process^66^. Through this method, the significance threshold for *R* ^2^(n, s) was estimated to be 0.8534. Coherence values outside the cone of influence (COI) were excluded for reliability (Fig. 1a). Crosstalk was also assessed at this stage - if high coherence (> 0.8534) was observed across a broad frequency range for most of the time, significant crosstalk was assumed, and the muscle pair was excluded from further analysis.

### Quantifying results from wavelet coherence analysis

IMC was calculated for the five muscle pairs (PL-TA, PL-SOL, PL-GM, SOL-TA, SOL-GM). The agonist (AG) and antagonist (ANT) muscle pairs are defined based on force action direction of muscle synergies^67^ (Table 2).

**Table 2:** Classification of agonist (AG) and antagonist (ANT) muscle Pair.

Muscle pairs	Inversion – Eversion	Dorsi-Plantar flexion
PL − TA	AG−ANT	-
PL − SOL	AG−ANT	-
PL − GM	AG−ANT	-
SOL − TA	AG− AG	AG−ANT
SOL − GM	AG−AG	AG−AG

(1) Phase category analysis – aims to identify patterns of temporal coordination. The significance threshold was applied to retain only high coherence values, which were categorised into four phase groups: in-phase (0 ± π/4), anti-phase (π ± π/4), phase shift of π/2 ± π/4, and opposite phase shift of -π/2 ± π/4. For each antagonist muscle pair $\:\mathrm{x}\left(n\right)$ – $\:\mathrm{y}\left(n\right)$, in-phase coherence reflects synchronous activation (muscle co-contraction), increasing joint stiffness and stability but limiting joint degrees of freedom and movement adaptability. In contrast, anti-phase coherence indicates muscle alternating activity as complementary antagonistic pairs, acting to fine-tune movement rather than stabilising through stiffness. Phase shift (π/2 ± π/4) suggests the reference muscle x(n) leads y(n), while opposite phase shift (-π/2 ± π/4) indicates a lag. The frequency (a count of occurrences) of each phase category across the frequency range of 0–64 Hz and time points was then calculated. The percentage of significant coherence $\:\mathrm{P}\left(s\right)$ within each phase category was calculated (Fig. 1b). The calculation of phase category at the group level $\:{P}_{Group}\left(s\right)$ was presented in Supplementary figure S1.

(2) Significant coherence share G, was computed for each condition (EO, EC) at the group level to qualitatively analyse the distribution of significant coherence, which was defined as:

$$\:\mathrm{G}=\frac{{\sum\:}_{i=1}^{{N}_{\mathrm{group}}}{\sum\:}_{t=1}^{{N}_{\mathrm{trial}}}{\sum\:}_{f\in\:{F}_{s}}{\sum\:}_{b\in\:{B}_{s}}{H}_{f,b}^{\left(i,t\right)}}{{\sum\:}_{i=1}^{{N}_{\mathrm{group}}}{\sum\:}_{t=1}^{{N}_{\mathrm{trial}}}{\sum\:}_{f}{\sum\:}_{b}{H}_{f,b}^{\left(i,t\right)}}\times\:100$$

A histogram of the coherence coefficients in the range from 0 to 1 was generated with a bin resolution of 0.01. *N*_group_ denotes the number of participants within a specific group (CAI or HC), and *N*_trial_ represents the number of trials that equal to three. $\:{H}_{f,b}^{\left(i,\:t\right)}$ represents the coherence count at frequency $\:\mathrm{f}$ and bin $\:\mathrm{b}$ for participant $\:\mathrm{i}$ in trial $\:\mathrm{t}$ within that group at a certain condition (EO or EC). $\:{F}_{s}$ represents the set of frequencies − 0.5 to 35 Hz (within the COI). $\:{B}_{s}$​ is the set of coherence values above the threshold for significance.

(3) The mean coherence M_((δ,β) ), for delta (0.5-5 Hz) and beta (15-35 Hz) band was computed for each participant. The frequency range of interest was chosen based on previous research examining IMC during human standing tasks^39^. The analysis was limited to frequencies ≥ 0.5 Hz because a 10-s window (Δf = 1/T = 0.1 Hz) contains five cycles at 0.5 Hz for reliable spectral estimates. For each frequency band, M was calculated by performing a weighted sum of histogram counts across coherence bins within the specified frequency range per participant:

$$\:{M}_{\left({\updelta\:},{\upbeta\:}\right)}=\frac{{\sum\:}_{b}\left({\mathrm{bin}\mathrm{\_}\mathrm{midpoint}}_{b}\cdot\:{\sum\:}_{f\in\:{F}_{\left({\updelta\:},{\upbeta\:}\right)}}{H}_{f,b}\right)}{{\sum\:}_{f\in\:{F}_{\left({\updelta\:},{\upbeta\:}\right)}}{\sum\:}_{b}{H}_{f,b}}$$

$\:{F}_{\left({\updelta\:},{\upbeta\:}\right)}$ denotes the mean coherence at delta and beta frequency range, bin_midpoint_b_​ is the centre value of per bin $\:\mathrm{b}$, $\:{H}_{f,b}$ denotes the coherence count at frequency $\:\mathrm{f}$ and bin $\:\mathrm{b}$ for each participant per trial. The values were then averaged across three trials and used for statistical analysis.

### Statistical analysis

To identify differences in mean coherence $\:{M}_{\left({\updelta\:},{\upbeta\:}\right)}$ between groups under balance conditions, repeated measures ANOVA was applied (2 groups (CAI and HC) × 2 conditions (eyes-open and eyes-closed)) to the mean coherence on five muscle pairs across two frequency bands, once the normal distribution of data was confirmed. Bonferroni post hoc tests were computed. Partial Eta Squared effect sizes were used to determine the magnitude of differences and were interpreted as small (0.01–0.06), moderate (> 0.06–0.14), and large (> 0.14)^68^. Linear multiple regression analysis was performed by using the forward stepwise method in SPSS (IBM SPSS 29, NY: IBM Corp): to investigate the correlation between mean IMC in the delta and beta frequency bands with COP-FD, analyses were conducted separately for eyes-open and eyes-closed conditions within each group.

## Results

### Qualitative analysis of significant coherence spectra

**Fig. 2:**
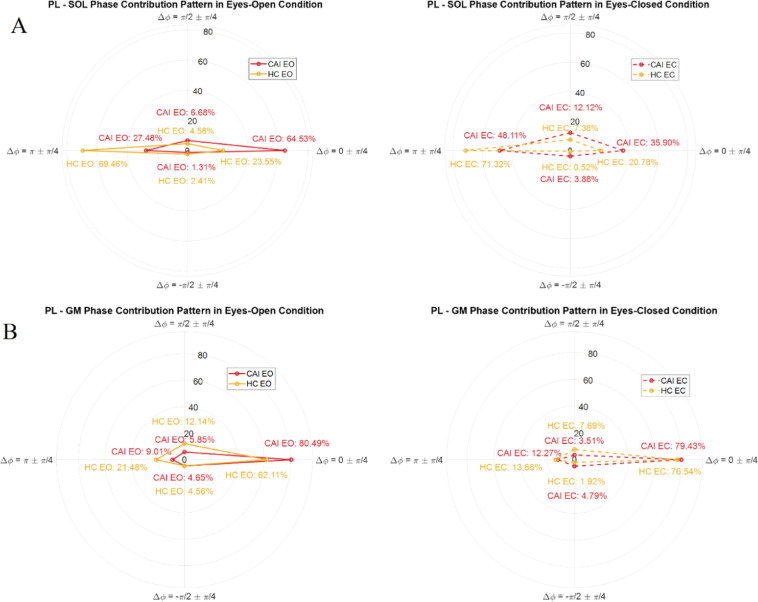
The figure compares the contribution patterns between chronic ankle instability (CAI) and healthy control (HC) groups in eyes-open (EO) and eyes-closed (EC) condition for PL-SOL (panel A) and PL-GM (panel B) muscle pairs, using four phase shift categories of significant coherence: Δφ = 0 ± π/4 (in-phase), π/2 ± π/4 (phase-shift), π ± π/4 (anti-phase), and − π/2 ± π/4 (opposite phase-shift). The percentages for each category represent the relative contribution of each phase shift condition within each group. The radial axis (y-axis) represents the contribution values (%), while the angular positions (x-axis) correspond to each phase shift condition. The red and yellow lines indicate the contributions for CAI and HC groups, respectively. The CAI group exhibits a pronounced tendency to shift coordination towards in-phase across both muscle pairs. Particularly, the PL-SOL muscle pair reveals that the CAI group exhibits a higher contribution of in-phase co-ordination, but less contribution of anti-phase compared to HC during eyes open (in-phase: 64.53 vs. 23.55%; anti-phase 27.48 vs. 69.46%).

For the PL-TA, SOL-TA, and SOL-GM muscle pairs, in-phase coherence dominates (approximately 90%) with similar patterns observed in both EO and EC conditions (Supplementary S1). The CAI group exhibits a tendency to shift coordination towards in-phase across PL-SOL and PL-GM muscle pairs (Fig. 2). Specifically, for the PL-SOL pair, the CAI group shows a greater in-phase contribution, and a reduced anti-phase contribution compared to the HC group during EO (in-phase: 64.53% vs. 23.55%; anti-phase: 27.48% vs. 69.46%). It suggests that individuals with CAI tend to rely more on co-contraction to stabilise the joint, whereas the HC group exhibits greater alternately activate as complementary antagonistic pairs.

**Fig. 3:**
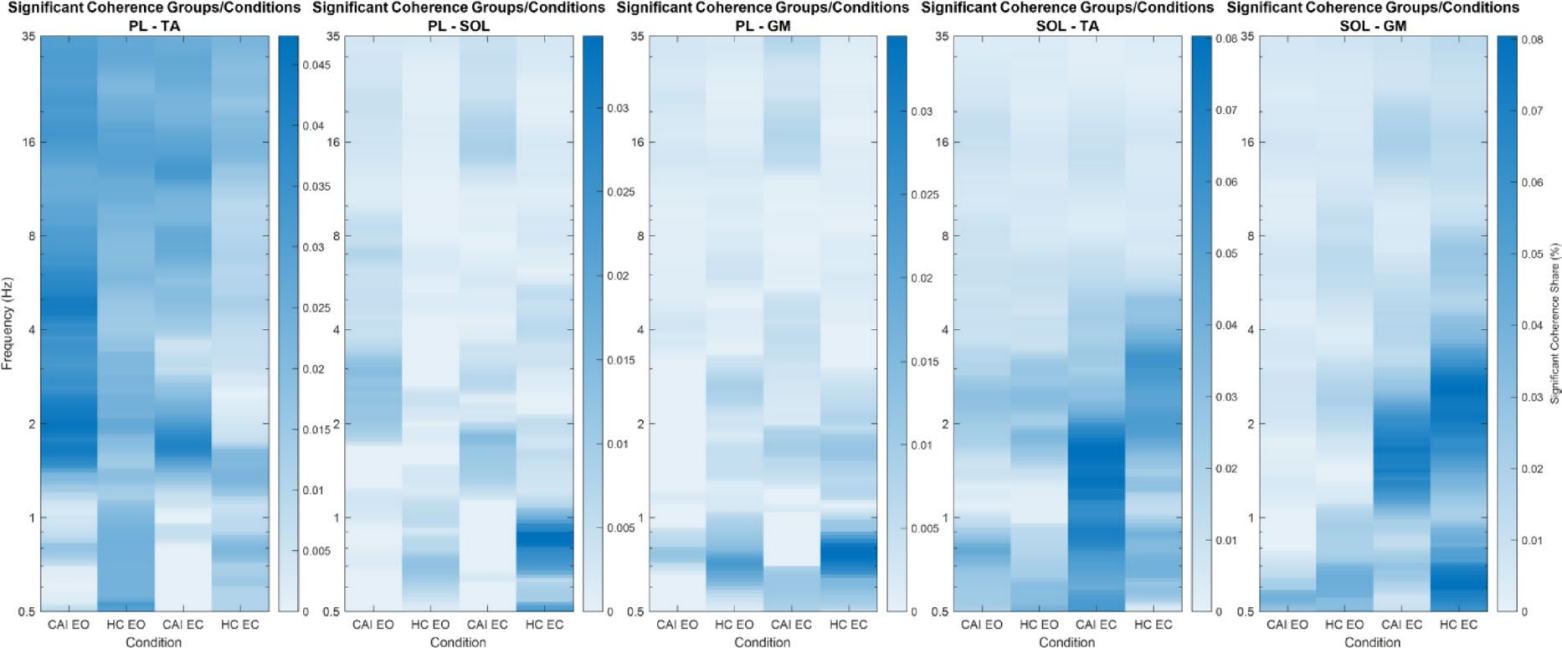
The heatmaps illustrate the five muscle pairs significant coherence share G (%) across different frequency bands (0.5 Hz to 35 Hz) for two groups (CAI and HC) during single leg standing under eyes open (EO) and eyes closed (EC) conditions. The x-axis represents the specified group and conditions, and the y-axis represents frequency bands on a logarithmic scale. The colour bar provides a reference for significant coherence share (%) values. Colour intensity reflects the proportion of significant coherence at the group level, with darker blue indicating a higher proportion of significant coherence (stronger muscle synchronisation) and lighter blue indicating a lower proportion (weaker synchronisation).

Figure 3 illustrates an increased proportion of significant coherence share in both groups under EC compared to EO, as indicated by higher density of significant coherence within the 0.5–5 Hz for SOL-TA and 0.5–35 Hz for SOL-GM pair. Additionally, the CAI group exhibited greater significant coherence share compared to the HC group across the beta-band (15–35 Hz) in PL-TA, PL-SOL, PL-GM and SOL-TA. Visual removal appeared to enhance coupling in CAI at beta-band frequencies, but the HC group did not exhibit a similar strengthening, indicating a distinct pattern of muscle coordination between the groups.

### Quantitative group mean coherence within pre-defined bands

Table 3 shows the weighted mean coherence in five muscle pairs within the delta (0.5–5 Hz) and beta (15–35 Hz) frequency bands. A significant interaction effect was found for delta Peroneus Longus (PL) - Tibialis Anterior (TA) (*F*_(1,30)_ = 6.986, *p* = 0.013) and beta PL - Gastrocnemius medial head (GM), (*F*_(1,30)_ = 5.688, *p* = 0.024), along with a group effect on beta PL-GM (*F*_(1,30)_ = 4.282, *p* = 0.047) and a condition effect on delta Soleus (SOL)-TA, (*F*_(1,30)_ = 15.797, *p* < 0.001), delta- (*F*_(1,30)_ = 77.121, *p* < 0.001) and beta-band (*F*_(1,30)_ = 70.541, *p* < 0.001) coherence in SOL-GM. Post hoc analysis demonstrated that during eyes-closed, the CAI group had greater beta-band coherence in PL-SOL (*p* = 0.047), PL-GM (*p* = 0.015) and SOL-TA (*p* = 0.048) compared to the HC group. Vision removal strengthened delta-band coherence in SOL-TA (*p* = 0.01) and both delta- and beta-band coherence in SOL-GM in both groups (*p* < 0.001). However, removing vision significantly reduced delta-band coherence in PL-TA in the CAI group only (*p* = 0.022).

### Postural sway

No significant main effect of group in COP-FD was found (CAI: 1.67 ± 0.08; HC: 1.70 ± 0.12, *p* = 0.448), nor was there a significant interaction effect, *F*_(1,30)_ = 0.682, *p* = 0.415. A marginal main effect of condition was observed (EO: 1.67 ± 0.11; EC: 1.70 ± 0.09, *F* (1,30) = 4.056, *p* = 0.053). Post hoc analysis indicated a statistical trend toward increased COP-FD in the HC group under the EC compared to EO (mean difference: 0.047, 95% CI: -0.001 to 0.094, *p* = 0.054).

### Relationships between intermuscular coherence and postural complexity

**Fig. 4:**
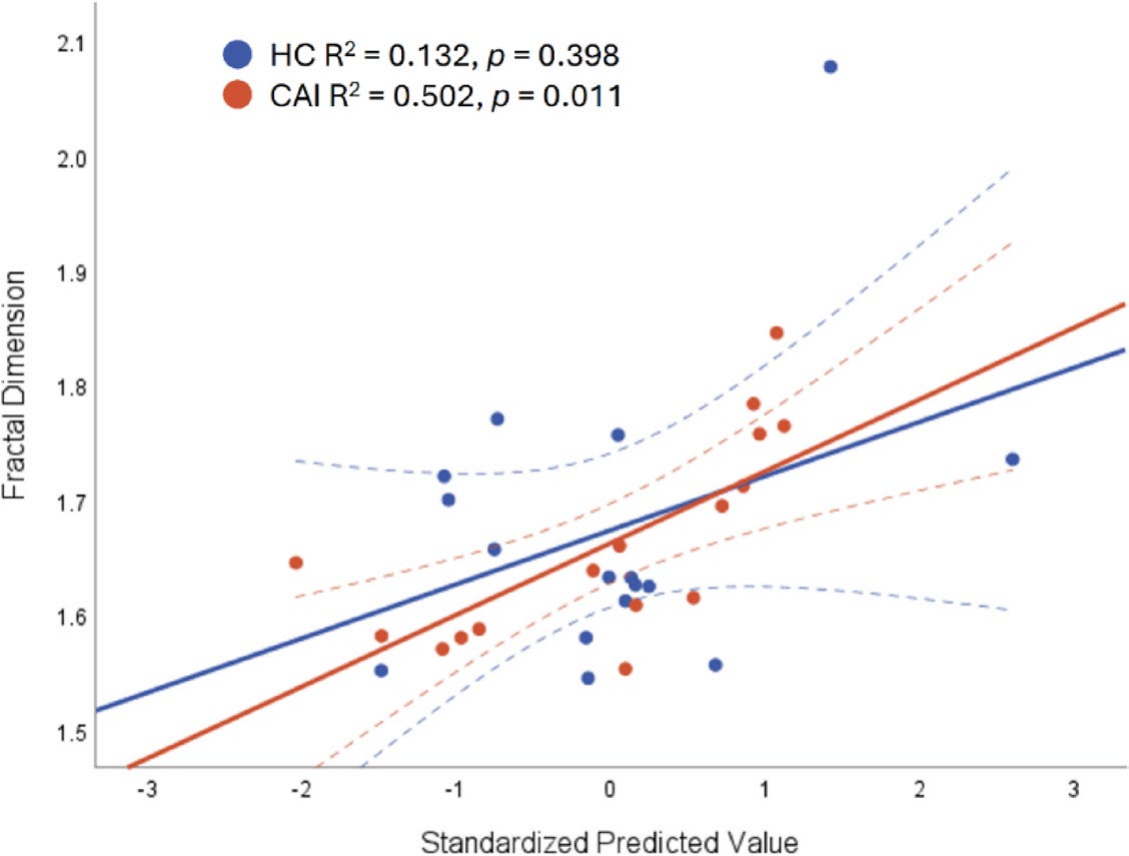
This figure illustrates the relationship between the Standardized Predicted Value of two independent variables (i.e. Delta PL – GM and Beta PL – GM) and the fractal dimension of centre of pressure for two groups (CAI and HC). The linear fit, with 95% confidence interval shows how well the model explains the relationship between the variables, R² and p value indicating the strength of the correlation.

Regression analysis was performed on each group separately, to assess whether IMC levels across delta and beta bands could predict postural complexity. Significant findings during eyes-open task were observed in CAI group (Fig. 4), that 50.2% of the variance in COP-FD could be explained by delta- and beta- band coherence in PL-GM (F _(2,15)_ = 6.563, *p* = 0.011), while this muscle pair showed no significant relationship in the HC group. The regression revealed that higher delta-, with lower beta-band coherence in PL-GM correlates with greater COP complexity. In contrast, no associations were found between mean coherence for any muscle pair and COP-FD in the HC group during eyes open. During eyes-closed task, a significant correlation was observed in both groups. Lower beta-band coherence in PL-TA correlates with more complex COP in CAI (R^2^ = 0.371, *p* = 0.012) and HC group (R^2^ = 0.375, *p* = 0.012).

Result Figures/tables

**Table 3:** Mean coherence (Mean ± SD) for the CAI and HC during one-leg static standing during eyes open (EO) eyes closed (EC) condition.

Muscle pair	Frequency band	Group												*P* values (partial eta squared)					
		CAI						HC						Interaction		Group		Condition	
		Eyes open			Eyes closed			Eyes open			Eyes closed			*p*	ηp^2^	*p*	ηp^2^	*p*	ηp^2^
PL - TA	Delta	**‡ 0.43**	**±**	**0.15**	0.35	±	0.14	0.32	±	0.16	0.36	±	0.10	**0.013**	**0.19**	0.297	0.04	0.449	0.019
	Beta	0.40	±	0.11	0.40	±	0.09	0.36	±	0.10	0.34	±	0.11	0.641	0.01	0.167	0.06	0.656	0.007
PL - SOL	Delta	0.36	±	0.08	0.35	±	0.07	0.35	±	0.09	0.39	±	0.10	0.192	0.06	0.532	0.01	0.409	0.023
	Beta	0.26	±	0.07	**†0.28**	**±**	**0.07**	0.24	±	0.03	0.24	±	0.04	0.158	0.07	0.092	0.09	0.23	0.048
PL - GM	Delta	0.33	±	0.08	0.34	±	0.07	0.33	±	0.11	0.37	±	0.08	0.528	0.01	0.559	0.01	0.194	0.056
	Beta	0.27	±	0.05	**†0.29**	**±**	**0.06**	0.25	±	0.04	0.24	±	0.05	**0.024**	**0.16**	**0.047**	**0.13**	0.751	0.003
SOL - TA	Delta	**‡ 0.44**	**±**	**0.11**	0.51	±	0.14	**‡ 0.44**	**±**	**0.10**	0.51	±	0.12	0.838	0.00	0.969	0.00	**< 0.001**	**0.345**
	Beta	0.31	±	0.07	**†0.33**	**±**	**0.06**	0.28	±	0.04	0.29	±	0.04	0.732	0.00	0.07	0.11	0.154	0.067
SOL - GM	Delta	**‡ 0.37**	**±**	**0.11**	0.51	±	0.10	**‡ 0.42**	**±**	**0.11**	0.56	±	0.11	0.687	0.01	0.162	0.06	**< 0.001**	**0.72**
	Beta	**‡ 0.32**	**±**	**0.05**	0.38	±	0.06	**‡ 0.32**	**±**	**0.05**	0.37	±	0.06	0.924	0.00	0.882	0.00	**< 0.001**	**0.702**

^†^Denotes significant difference between CAI the HC (*p* < 0.05). Bonferroni adjusted for multiple comparisons.

^‡^Denotes significant difference between EO and the EC conditions (*p* < 0.05). Bonferroni adjusted for multiple comparisons.

Effect Size was estimated by Partial Eta squared (ηp^2^). Tibialis Anterior (TA), Peroneus Longus (PL), Gastrocnemius medial head (GM), and Soleus (SOL). Delta band: 0.5–5 Hz; Beta band: 15–35 Hz.

## Discussion

The study investigated the effects of visual input on IMC in individuals with CAI compared to healthy controls for the five muscle pairs (PL-TA, PL-SOL, PL-GM, SOL-TA, SOL-GM) during static single-leg stance. The main findings were as follows. Individuals with CAI had different patterns of IMC, particularly on agonist (AG) - antagonist (ANT) muscle pair during eyes-closed. The CAI group had greater beta-band coherence during eyes closed in PL-SOL, PL-GM and SOL-TA compared to the HC group. This suggests that the CAI group have greater synchronisation of motor unit activity driven by descending supraspinal commands through corticospinal drive to antagonistic muscle pairs. There was no group difference in delta-band coherence in any muscle pair or postural complexity, rejecting our hypothesis. Higher beta-band coherence in the antagonistic muscle pairs correlated with reduced COP complexity in both groups, suggesting that strengthened beta-band indicates reduced postural adaptability.

The current study found that individuals with CAI exhibited greater beta-band agonist-antagonist IMC during eyes-closed compared to the HC group, suggesting the CAI may adopt a corticospinal adaptation strategy when visual information was removed. Beta-band coherence has been shown to reflect corticospinal drive to -motoneurons innervating muscles^37^, and includes a strong supraspinal component, as reduced coherence has been observed in individuals with stroke^69^ and spinal cord injury^70,71^ during muscle contraction. Previous studies have shown that older adults display higher beta-band coherence in the MG-SL pair during single-leg tasks compared to younger adults^72^. During walking, older adults demonstrate greater beta-band coherence in rectus femoris to biceps femoris, gluteus medius to adductor magnus muscle pairs, and strengthened cortico-muscular coherence, reflecting proximal adaptation strategies^40^. Increased beta-band coherence might suggest less efficient corticospinal transmission in CAI individuals. Specifically, increased beta-band coherence may reflect a less heterogeneous population of motoneurons^73^, where reduced variability in thresholds and properties of motoneurons limits asynchronous sampling, impairing efficient motoneuron discharge^73,74^.

Decreased signal transmission efficiency limits the ability descending motor command to muscles, leading to reduced fine-tune movement and postural complexity. This speculation was confirmed by the association between higher beta-band coherence in the PL-TA pair with reduced COP complexity in the current findings. Increased beta coherence was associated with higher COP variability and velocity during standing, suggesting a link between beta synchronisation and postural control demands^72^. Stronger beta-band cortico-muscular coherence stabilises corticospinal interactions to compensate for dynamic modulated forces when higher force level is required^75^. Given this physiological basis, the current findings imply that individuals with a more rigid, less adaptable postural control strategy rely on stronger beta-band synchronisation to modulate dynamic forces and compensate for reduced responsiveness to postural challenges. Previous studies have shown that individuals with acute ankle sprain demonstrated reduced COP-FD during eyes-closed single-leg stance compared to uninjured individuals, with even lower COP-FD observed in those unable to complete the task, indicating a postural control system less able to fulfil the demands of the task^62^. Additionally, individuals with CAI exhibited lower COP-FD than healthy controls^61^. COP-FD has been associated with changes in postural stability^76,77^, pathology^78,79^ and sensorimotor activity during balance^79,80^, suggesting that COP-FD might reflect the activity of the postural control system. In the current study, increased COP-FD with eyes-closed was observed only in the HC group, supporting the idea that individuals with CAI may have deficits in upregulating postural control activity to meet higher task demands, limiting available sensorimotor redundancies. It should be noted that other analyses such as COP sway path length, or COP measures correlated with EMG amplitude, may provide different insights but were beyond the scope of this study. The observed association between IMC and COP-FD supplements that elevated beta-band coherence may indicate reduced postural control system activity. While the origin of beta-band input is unclear, this exploratory study showed increased beta-band coherence, suggesting that reduced corticospinal transmission efficiency might contribute to lower postural control adaptability in CAI. The dysfunctional beta-band coherence shows promise as a non-invasive biomarker for identifying neuromuscular deficits in CAI, though further studies are needed to confirm this. Excluding CAI copers limits our understanding of the full spectrum of individuals with past ankle sprains but no residual symptoms, particularly whether their corticospinal inputs have returned to levels similar to healthy controls. If so, interventions like transcranial direct current stimulation^81^, and electrical stimulation^82^ may be effective. Alternatively, if copers display similar corticospinal responses to those with CAI, the functional importance of coherence changes may be questioned.

Vision removal primarily increased delta- and beta-coherence in agonist-agonist muscle pairs in both groups. This is consistent not only with studies showing increased coherence in AG-AG pairs when vision is removed^44^, but also when task demands are increased, such as transitioning from two-leg to single-leg stance^43,72^. This increase suggests that enhanced balance difficulty promotes co-contraction for greater torque rather than synergistic activation in agonist-antagonist pairs for joint stiffness. Contrary to our hypothesis, current study did not observe group differences in delta-band coherence in any muscle pair. Vision removal significantly decreased delta-band coherence in the PL-TA pair in the CAI group, highlighting potential muscle imbalance. Individuals with CAI often exhibit reduced proprioceptive feedback from Ia afferents^4^, and decreased PL reflex excitability^18^. These deficits may contribute to disruption of the normal reciprocal inhibition pattern between PL and TA muscles^83,84^, which typically inhibit PL while overactivity of the TA, disrupting balance between these muscles, but there is no direct evidence of altered reciprocal inhibition in CAI.

A significant association between delta- and beta-band coherence in the PL-GM pair and COP-FD was observed in the CAI group during the eyes-open condition, but not in the HC group. Further analysis on this muscle pair showed that, during eyes-closed, COP-FD was predicted by beta-band PL-GM coherence in the CAI with no significant association in the HC group. This pattern suggests that CAIs may rely on greater active neuromuscular control to maintain balance, which might be due to increased joint laxity^4^, compared to healthy participants, who mainly relies on intrinsic stiffness during quiet standing^28^. Low-frequency oscillations are related to force generation and variability^38,41^, which possibly compensate for impaired ankle strength in individuals with CAI. It is noted however, that this interpretation has been argued by Dideriksen, Negro, Falla, Kristensen, Mrachacz-Kersting and Farina^63^, because common input may not be accurately reflected in surface EMG due to distortions caused by variability in motor unit action potential shapes^34,36^. Specifically, IMC is weakly correlated with motor unit coherence at frequencies lower than 5 Hz^63^, because the summation of biphasic action potentials causes amplitude cancellation, leaving the composite EMG signal are weakly correlated with the synaptic inputs received by the motor neurons^85^. Another consideration is the link between IMC and the neural input might be valid only at low forces, typically below 10% of maximal voluntary contraction (MVC)^63^, yet peroneus muscle activity during single-leg stance averages roughly 21 ± 9% MVC^86^. Future research is warranted to validate the association between IMC and neural input at higher levels of force. Despite these arguments, our exploratory investigation, building on previous studies^42,43,72^, further demonstrates the functionality of IMC to postural control, bridging the relationship between beta- and delta-band IMC and CoP complexity during single-leg standing.

There are some limitations to be addressed. A key limitation of this study is the use of a 10-second analysis window, which may increase sensitivity to coherence fluctuations. The 10-second window was chosen to minimise muscle fatigue effects on synchronisation, as fatigue can decrease coherence^87^. The recording length of data required to robustly establish intermuscular coherence during single-leg stance, which has not been standardised or validated, with reported duration ranging from 40-s^72^, 30-s^43^ to 20-s^42^. Future work should assess the minimum signal length required for stable coherence. The anatomical proximity between PL and TA muscles does not fully rule out the potential for crosstalk, and no definitive mitigation methods are currently available^88^. However, we observed that Significant Coherence Share G across frequency bands was below 0.05% (Fig. 3), indicating that crosstalk was likely not significant. The electrodes were placed at two-thirds of the distance from the medial condyle of the femur to the medial malleolus, limiting our findings to this specific soleus compartment. Finally, detailed histories of ankle sprains and giving way could not be reported due to recall difficulties; future studies should include both retrospective and prospective tracking of ankle injuries to examine whether the identified neuromechanical markers predict sprain risk.

## Conclusion

Individuals with CAI exhibited increased corticospinal common inputs on agonist-antagonist muscle pairs during eyes-closed single-leg stance, but not in eyes-open conditions. People with CAI showed greater beta-band IMC for PL-SOL, PL-GM and SOL-TA during eyes-closed stance compared to healthy controls, suggesting increased corticospinal drive to these muscles. Higher beta-band coherence in the antagonistic muscle pairs correlated with reduced COP complexity in both groups, suggesting that strengthened beta-band indicates reduced postural adaptability. Dysfunctional beta-band IMC shows promise as a simple, non-invasive biomarker for identifying neuromuscular deficits in CAI, though further studies are needed to confirm this. This work is exploratory in nature; future research should validate the link between IMC and neural input at higher levels of force and the minimum signal length required for stable coherence. Future studies should include CAI copers to determine whether their corticospinal inputs have returned to healthy levels, supporting the potential effectiveness of interventions like transcranial direct current stimulation, or whether they display similar responses to those with CAI, which would question the functional significance of coherence changes.

## Supplementary Information

Below is the link to the electronic supplementary material.

Supplementary Material 1

## Data Availability

The datasets analysed during the current study are available from the corresponding author (XX) on reasonable request.
