# Supplementary material for: Increased common corticospinal input during eyes-closed unilateral stance in people with chronic ankle instability
Source: Sci Rep. 2026 Feb 12;16:8525. doi: 10.1038/s41598-026-39425-3 (PMC12976301; doi:10.1038/s41598-026-39425-3)
Supplement: Supplementary file 1 — Supplementary Material 1 [file 41598_2026_39425_MOESM1_ESM.pdf]

Supplementary

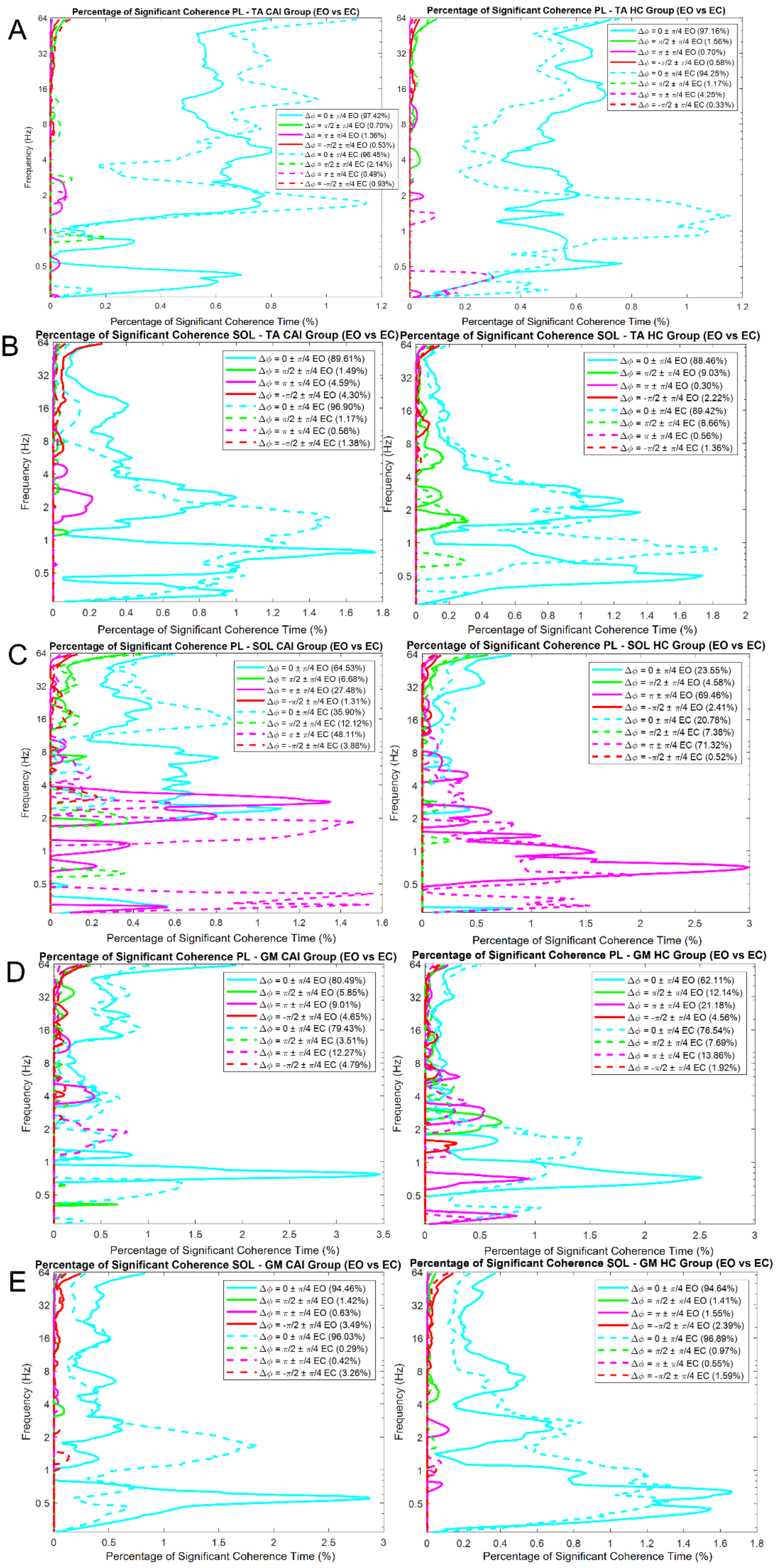

Figure S1. Percentage of significant coherence time across different frequency bands (0-64 Hz) for each muscle pair and group (CAI vs. HC) under eyes open (EO) and eyes closed (EC) conditions. Panels (A) to (E) display the coherence time distribution across four phase categories: The percentage of significant coherence was quantified within four distinct phase ranges: $\Delta\phi$ = 0± π/4 (in-phase, cyan), π/2 ± π/4 (phase-shift green), π ± π/4 (anti-phase purple), and −π/2 ± π/4 (opposite phase-shift red).

$P_{groups}(s)$ was computed by taking the count of occurrences in each phase category at each frequency s, dividing by the total count of significant coherence occurrences for a certain condition (EO or EC) across participants within the group (CAI or HC). The cumulative percentage for each phase range is indicated in the legend.

For PL-TA (panel A), SOL-TA (panel B), and SOL-GM (panel E) muscle pairs, the in-phase coherence contributes the most (around 90%), showing similar patterns between eyes open (EO) and eyes closed (EC) conditions. However, for PL-SOL (panel C) and PL-GM (panel D) pairs, both in-phase and anti-phase contributions are prominent. Visual removal altered the phase contribution pattern in PL-SOL pairs, particularly in the CAI group, where in-phase coherence decreases from 64% to 36%, while anti-phase coherence increases from 27% to 48%.
